# Frequency of Infectious Mortality at the End of Induction Chemotherapy in Acute Lymphoblastic Leukemia and Lymphoma Patients: Findings From a Tertiary Care Cancer Center

**DOI:** 10.7759/cureus.13208

**Published:** 2021-02-07

**Authors:** Rabia Wali, Sadia Anjum, Asim Amjad, Najma Shaheen, Saadiya Javed Khan

**Affiliations:** 1 Paediatric Oncology, Shaukat Khanum Memorial Cancer Hospital and Research Centre, Lahore, PAK; 2 Paediatrics, Mayo Hospital, Lahore, PAK; 3 Paediatrics and Child Health, King Faisal Specialist Hospital and Research Centre, Riyadh, SAU

**Keywords:** acute leukemia, induction chemotherapy, lymphoblastic lymphoma, mortality

## Abstract

Background and objective

In low- and low-to-middle-income countries (LMICs), the incidence of treatment-related mortality (TRM) in patients with acute lymphoblastic leukemia (ALL) and lymphoblastic lymphoma (LBL) is up to 52%. This study aimed to determine the mortality rate at the end of the induction phase of the treatment among patients with ALL and lymphoma at a tertiary care cancer center.

Methods

This retrospective study analyzed outcomes after induction chemotherapy in pediatric patients with acute leukemia and lymphoma at a tertiary care cancer center from January 2015 to December 2016. Information regarding demographics, clinical characteristics, and laboratory investigations were extracted and reviewed.

Results

Of the total 160 patients, 110 were males, and the mean age of the sample was 4.6 +2.8 years. B-cell leukemia (pre-B-ALL) was diagnosed in 84% (n=134), while 10% (n=6) had acute T-cell leukemia (pre-T-ALL) and 6% (n=10) had lymphoma. Sixteen patients (10%) died within the defined induction period, with 14 deaths occurring due to infections and two deaths resulting from chemotherapy-related toxicity.

Conclusion

Based on our findings, there is a significant prospect of mortality from infections during induction chemotherapy in patients with pediatric hematological malignancies.

## Introduction

Acute lymphoblastic leukemia (ALL) and lymphoblastic lymphoma (LBL) are among the most common hematological malignancies among pediatric patients and account for approximately 30% of all childhood cancers. In the last 30 years, advances made in treatment have led to five-year survival rates of more than 80% [1]. This has been accomplished through a better understanding of tumor biology at a molecular level, risk assignment, and intensification of chemotherapeutics based on a risk-adapted approach. Furthermore, improvements in supportive care strategies such as early detection and prompt treatment of infections, optimization of blood product administration, and early intensive care (ICU) referrals have contributed to better outcomes [2,3]. Nonetheless, children may still experience extremely toxic drug-induced side effects, which can even result in treatment-related mortality (TRM). The majority of these reactions occur during the initial induction phase of the treatment [4].

The incidence of TRMs due to infections in patients with acute leukemia and lymphoma is about 2-4% [5]. However, the TRM percentage in low-to-middle-income countries (LMICs) is much higher, and TRM incidence of up to 52% has been reported [6-8]. This disparity in rates is likely due to infections, suboptimal supportive care, malnutrition, chemotherapy-related toxicity, delay in diagnosis, and abandonment due to social issues [9].

The primary aim of this study was to determine the mortality rate at the end of the induction phase of the treatment in patients with ALL and LBL at a tertiary care cancer center operating in an LMIC.

## Materials and methods

We conducted a retrospective study on pediatric ALL and LBL patients treated at the Shaukat Khanum Memorial Cancer Hospital (SKMCH) between January 2015 and December 2016. All patients with leukemia and lymphoma treated with a standard chemotherapeutic regimen were included in the study. However, patients of less than one year of age or those with mature B- and T-cell lymphoma were excluded. The Institutional Review Board at SKMCH approved the study and gave the go-ahead to collect data on patient demographics, clinical features, pathology, imaging, treatment, and outcomes from the hospital's electronic medical records (approval number: EX-01-08-17-02-A1).

The patients were risk-stratified according to the National Cancer Institute (NCI) guidelines and treated based on the United Kingdom Acute Lymphoblastic Leukemia (UKALL) 2013 guidelines [10]. Cranial (CNS) disease was defined as the presence of lymphoblasts on cytospin preparation at the time of diagnostic lumbar puncture. Patients between 1-9.99 years of age, those with a white cell count of less than 50,000, or those with precursor B-cell immunophenotype were stratified as standard risk (SR). On the contrary, patients were categorized as high risk (HR) if they were above 10 years of age, if their white cell count was more than or equal to 50,000, or if they had precursor T-cell immunophenotype and LBL. The SR patients received a three-drug induction for four weeks with dexamethasone 6mg/m^2^ for 28 days, vincristine weekly, and PEGylated asparaginase in two doses. The HR patients received a four-drug induction with the addition of daunorubicin weekly to the above-mentioned three-drug regimen. All patients received intrathecal (IT) methotrexate (MTX) on day one and subsequent weekly ITs based on CNS involvement.

All newly diagnosed ALL and LBL patients were admitted to the hospital for the initiation of the treatment. They were managed for tumor lysis prophylaxis for the first 24-48 hours, which was followed by chemotherapy. Bone marrow re-assessment was done on days eight and 15 based on risk stratification and then at end of the induction on day 29 in all patients for disease remission. If patients were clinically well after day four of the induction chemotherapy, they were discharged and then were subsequently seen every week in the outpatient clinic.

While on induction therapy, all patients were advised to come to the emergency room if they had a fever. Fever was defined as a single instance of a temperature of at least 38 °C within the last one hour or a single spike of 38.3 °C [4]. In the emergency room, all patients with fever underwent a thorough history and examination and had complete blood count, blood cultures, and urine culture or chest imaging, wherever clinically appropriate. Furthermore, a stat dose of broad-spectrum antibiotics was administered. Patients were hospitalized and clinically presumed to have an infection if the absolute neutrophil count was less than 1,000 in the absence of positive cultures.

Diagnosis of infection was validated with microbiologically proven blood cultures, urine cultures, tissue specimens from the chest or fine needle aspirations, or endoscopically from core biopsy of sinuses. Blood cultures were taken on admission and then repeated in cases of new fever, change of antibiotics, and daily in case of persistent fevers. The causes of death were categorized as infection-associated, treatment-related toxicity, or bleeding complications. The duration of the induction was 42 days from the first day of admission including the pre-induction phase, and all deaths that occurred during this period were included in this analysis.

All the collected data were analyzed using Microsoft Excel version 10 (Microsoft Corporation, Redmond, WA) and SPSS Statistics version 19 (IBM, Armonk, NY). Descriptive statistics were applied to calculate the mean and standard deviation. Frequency distribution and percentages were calculated for qualitative variables using a sample t-test. Overall, a p-value of less than 0.05 was considered statistically significant.

## Results

A total of 160 patients were included in this study. The mean age of the sample was 4.6 +2.8 years, and more than 90% of the patients were less than five years of age. The male-to-female ratio was 2.2:1. The most common primary disease was B-cell leukemia (pre-B-ALL; 83.8%), and most of the participants were categorized as low risk (76.9%). During the defined induction period, 16 patients (10%) died (Table 1). Among these, 14 (87.5%) deaths were infection-associated, and two (12.5%) were from chemotherapy-related toxicity. Gastrointestinal manifestations were seen in four patients (25%), while five (32%) had respiratory symptoms along with positive cultures and seven (43%) patients had bacteremia.

Infections were microbiologically verified in 12 (85%) patients. In these 12 patients with a pathologically proven bacterial infection, there were a total of 16 positive cultures: nine positive blood cultures, two tracheal aspirates, two endoscopic sinus biopsies, and one each for peritoneal fluid, CT-guided lung biopsy, and ear swabs were isolated.

Nine cultures (56%) were positive for gram-negative bacteria (GNB), and among these, five cultures had multidrug-resistant *Escherichia coli* (MDR-*E. coli*), three had pan-sensitive *E.coli*, and one sample came back positive for *pseudomonas*. Similarly, in five subjects, five (31%) samples tested positive for fungal infection. Three subjects with positive fungal cultures also had positive bacterial cultures. Two tracheal aspirates and a lung nodule grew fungal *Aspergillus*, and *Mucor* grew from one endoscopic biopsy and *Fusarium*from the ear discharge (Table 2).

**Table 1:** Characteristics of leukemia and lymphoma patients B-ALL: B-cell acute lymphoblastic leukemia; T-ALL: T-cell acute lymphoblastic leukemia; LBL: lymphoblastic lymphoma

Patient parameters	N (%), total=160
Age	
1-5 years	147 (92%)
≥5 years	13 (8%)
Gender	
Male	110 (69%)
Female	50 (31%)
Primary disease	
Pre-B-ALL	134 (84%)
Pre-T-ALL	16 (10%)
T-/B-LBL	10 (6%)
Risk group	
Low risk	123 (77%)
High risk	37 (23%)
Infections	
Yes	14 (8.75%)
No	146 (91.25%)
Outcomes	
Alive	144 (90%)
Dead	16 (10%)

**Table 2:** Positive cultures from all sites MDR: multidrug-resistant; *E. coli: Escherichia coli*

Patients (n=12)	Organisms cultured	Positive cultures/site (n=16)
1	Aspergillus	Tracheal aspirate
2	MDR *E.coli*	Blood CS
3	MDR *E.coli*	Blood CS
4	MDR *E.coli*	Blood CS
5	E.coli	Blood CS
6	E.coli	Peritoneal fluid
7	MDR E.coli	Blood CS
	Mucor	Endoscopic biopsy from sinus
8	Fungal hyphae	CT chest nodule biopsy
9	Pseudomonas	Blood CS
10	E.coli	Blood CS
	Aspergillus	Tracheal aspirate
11	Vancomycin-resistant *Enterococci*	Endoscopic biopsy from sinus
	Fusarium	Ear swab
12	MDR *E.coli*	Blood CS
	Vancomycin-resistant *Enterococci*	Blood CS

One of the two toxicity deaths was secondary to pancreatitis leading to disseminated intravascular coagulation (DIC). The other was related to vincristine-induced paralytic ileus leading to a mechanical obstruction that resulted in bowel perforation. All deaths occurred between 15 to 24 days of the induction phase. Bone marrow was in remission on day 29 in 136 patients (85%). Of the 16 subjects who died, bone marrow examination was possible in only two patients, and both were in remission.

## Discussion

Favorable outcomes related to ALL and LBL in LMICs remain low despite improvements in supportive care services. TRM, primarily due to infections, remains one of the significant factors affecting outcomes. Notably, a high number of deaths occurs during the early induction phase. This study aimed to determine the mortality rate at the end of the induction among patients with leukemia and lymphoma at a tertiary care cancer center operating in an LMIC. The incidence of induction mortality in this study was 10%. Of these, 87.5% of the deaths were related to infections, and 12.7% were secondary to chemotherapy-related toxicity.

Studies from various LMICs have reported induction death rates ranging from 12.8 to 22.6% [11,12]. In comparison, in prior retrospective analyses from the center where the present study was conducted, the induction death ranged from 24 to 52% [8,13]. In the present study, TRM was found to be 10%. This improvement in outcome is likely associated with the developments in infrastructural facilities, quality and availability of blood products, and better infection control practices and treatment measures. Nonetheless, this rate is still three to four times higher than the rates reported in high-income countries [14].

In the present study, 87.5% of the induction deaths were associated with infections, and among 16 positive cultures, 56% were associated with GNB. These results are similar to those reported by Fadoo et al. [6]. Investigators found that 63.5% of the 52 samples of blood cultures were significant for GNB. Generally, it appears that there is a low percentage of positive cultures of gram-positive bacteria. The exact reason for this is unknown and beyond the scope of this manuscript. However, it could be secondary to a lack of central line placement in this cohort of patients.

Fungal infections pose a significant threat to children with ALL and lymphomas. Previously, the rate of invasive fungal infections among patients with hematological malignancies was reported to be between 1.3-25% [15,16]. However, in the present study, the fungal infection rate was 31%, and among these, three subjects had concurrent positive cultures for bacteremia. During the induction phase, patients develop neutropenia and are immunocompromised, making them susceptible to bacterial and fungal infections. However, this alone may not explain why nearly one-third of the infections were fungal in origin in the present study. A possible contributing factor could be the onsite construction during the study period. Multiple reports in the literature suggest an association between invasive fungal infections and contaminated ventilation systems, hospital construction, or renovations [17,18].

Seven (44%) out of the 16 bloodstream infections were MDR *E.coli* and vancomycin-resistant *Enterococci*. This high percentage could be due to the particular characteristics of the cohort of patients who are treated at our center. The majority of the patients are referred to from low-income communities, where indiscreet use of antibiotics is common for minor ailments (fever, flu, and respiratory or gastrointestinal symptoms). Furthermore, due to the inherent nature of hematological malignancies, these patients frequently experience fever, which is routinely managed locally with broad-spectrum intravenous antibiotics.

A limitation of this study is that it was a retrospective analysis. Hence, it was impossible to have an equal distribution of subjects with SR and HR, central line access in all subjects, or control for other clinical and management-associated characteristics. Moreover, due to the nature of the research design, there was a lack of randomization and blindness, resulting in a high likelihood of false-positive associations or magnification of positive responses. Nonetheless, data were extracted from an electronic database and verified by reviewing follow-up visits.

## Conclusions

In conclusion, there is a significant prospect of mortality associated with induction chemotherapy among patients with ALL and lymphoma. A significant number of deaths in our study were secondary to infections. Infections remain a major healthcare challenge in LMICs. Raising awareness about the resistance caused by excessive antibiotic usage can further help decrease our mortality rates. Going forward, we intend to examine the prophylactic antibiotic use during induction therapy among our patients in a prospective manner.
